# Functional analysis of *POLD1* p.ser605del variant: the aging phenotype of MDPL syndrome is associated with an impaired DNA repair capacity

**DOI:** 10.18632/aging.202680

**Published:** 2021-02-22

**Authors:** Michela Murdocca, Paola Spitalieri, Claudia De Masi, Ion Udroiu, Jessica Marinaccio, Massimo Sanchez, Rosa Valentina Talarico, Chiara Fiorillo, Monica D’Adamo, Paolo Sbraccia, Maria Rosaria D’Apice, Giuseppe Novelli, Antonella Sgura, Federica Sangiuolo

**Affiliations:** 1 Department of Biomedicine and Prevention, Tor Vergata University, Rome 00133, Italy; 2 Department of Science, “Roma Tre” University, Rome 00154, Italy; 3 Department of Cell Biology and Neurosciences, Istituto Superiore di Sanità, Rome 00161, Italy; 4 Paediatric Neurology and Neuromuscular Disorders, University of Genoa and Istituto Gaslini, Genoa 16147, Italy; 5 Department of Systems Medicine, Tor Vergata University, Rome 00133, Italy; 6 Laboratory of Medical Genetics, Tor Vergata Hospital, Rome 00133, Italy

**Keywords:** MDPL syndrome, *POLD1* gene, age-related disease, DNA repair, telomere damage

## Abstract

Mandibular hypoplasia, Deafness and Progeroid features with concomitant Lipodystrophy define a rare systemic disorder, named MDPL Syndrome, due to almost always a *de novo* variant in *POLD1* gene, encoding the DNA polymerase δ.

We report a MDPL female heterozygote for the recurrent p.Ser605del variant. In order to deepen the functional role of the in frame deletion affecting the polymerase catalytic site of the protein, cellular phenotype has been characterised. MDPL fibroblasts exhibit *in vitro* nuclear envelope anomalies, accumulation of prelamin A and presence of micronuclei. A decline of cell growth, cellular senescence and a blockage of proliferation in G0/G1 phase complete the aged cellular picture. The evaluation of the genomic instability reveals a delayed recovery from DNA induced-damage. Moreover, the rate of telomere shortening was greater in pathological cells, suggesting the telomere dysfunction as an emerging key feature in MDPL.

Our results suggest an alteration in DNA replication/repair function of *POLD1* as a primary pathogenetic cause of MDPL.

The understanding of the mechanisms linking these cellular characteristics to the accelerated aging and to the wide spectrum of affected tissues and clinical symptoms in the MDPL patients may provide opportunities to develop therapeutic treatments for progeroid syndromes.

## INTRODUCTION

Mandibular hypoplasia, Deafness and Progeroid features with concomitant Lipodystrophy, represent a rare systemic disorder, named MDPL syndrome (MDPL; OMIM #615381) with a prevalence <1/1,000,000. MDPL was described for the first time in 2010 [1], reporting seven subjects showing a clinical phenotype overlapping with mandibuloacral dysplasia syndromes (MADA and MADB) such as mandibular hypoplasia, prominent eyes, stiff joints, beaked nose, and lipodystrophy, but also specific additional clinical hallmarks, including sensorineural hearing loss, hypogonadism and absent clavicular hypoplasia/acroosteolyses.

MAD and MDPL belong to the group of diseases characterized by premature aging, which can be caused by inheritable nuclear envelope and/or DNA repair defects [2].

To date, only 26 patients with MDPL Syndrome have been described and all of them reported variants in *POLD1* gene (NM_002691.3) [3], encoding for the evolutionarily conserved p125 subunit of DNA polymerase delta (Polδ). It provides the essential catalytic activities of the enzyme, mediated by 5′–3′ DNA polymerase and 3′–5′ exonuclease moieties [4]. p125 subunit forms a heterotetramer with three smaller accessory subunits encoded by the *POLD2* (p50), *POLD3* (p66), and *POLD4* (p12) genes which, together with Replication Factor C and Proliferating Nuclear Cell Antigen, constitute the polymerase holoenzyme [5]. Both exonuclease and polymerase activities of Polδ are fundamental to the nuclear function of the enzyme. Most recently, a cytoplasmic function of Polδ has also been reported residing on the Golgi complex, where the Polδ controls microtubule growth [6]. Other studies have evidenced that Polδ also acts as a nucleocytoplasmic shuttling protein transported into and out of the nucleus in a controlled manner [7].

*POLD1* gene transcription is regulated throughout the cell cycle, where relatively small increases in mRNA levels occur in late G1/S phase, accompanied by corresponding modest increases in p125 protein levels [8]. Song and colleagues also demonstrated that *POLD1* downregulation is able to block the cell cycle at G1 and G2/M phases and results in reduced DNA synthesis [9], demonstrating the potential role of *POLD1* in the regulation of cell cycle progression.

Furthermore, evidence about Polδ activity has highlighted its fundamental involvement in DNA replication process, cooperating with a DNA helicase, WRN, to maintain genome stability [10]. Also the downregulation of p125 subunit is sufficient to induce genomic instability, culminating in DNA replication errors [11]. Polδ function is in fact essential for replication, with a primary role as the replicase for the lagging strand, but it also has an important proofreading ability conferred by the exonuclease activity, which is critical for ensuring replicative fidelity. Polδ serves to repair DNA lesions arising as a result of exposure to mutagens, acting in multiple forms of DNA repair, including nucleotide excision repair, double strand break repair, base excision repair, and mismatch repair [5]. During double strand breaks, cells can choose, as repair mechanism, between non homologous end joining (NHEJ) or homologous recombination (HR) process, depending on the type of lesion and the timing of the cell cycle. Sometimes, the instability of the holoenzyme can result in an increase in stalled or collapsed forks and the inability to quickly repair these breaks leads to genomic instability and apoptosis [12, 13]. Most importantly Polδ is involved in telomere break repair: in particular for the synthesis of both C- and G-rich telomere strands [14, 15].

Moreover, an inverse correlation between *POLD1* gene expression and age has been described both *in vitro* and *in vivo* [16], suggesting that *POLD1* may be associated with aging, but the functional link still remains unclear.

We report the *in vitro* characterisation of a known in-frame deletion p.Ser605del identified in a 22 years old Italian girl with clinical features of MDPL syndrome. In order to elucidate the functional role of this deletion, MDPL cellular phenotype has been characterised in terms of nuclear morphology, cellular proliferation, senescence, and cell cycle progression. In particular, we shed light on the capacity of MDPL cells to respond and repair DNA induced-damage, especially at telomeric level. The understanding of the pathogenic mechanism lying at the basis of the MDPL syndrome allows us to explore the link existing among DNA repair and age-related diseases.

## RESULTS

### Clinical phenotype

**Figure 1:**
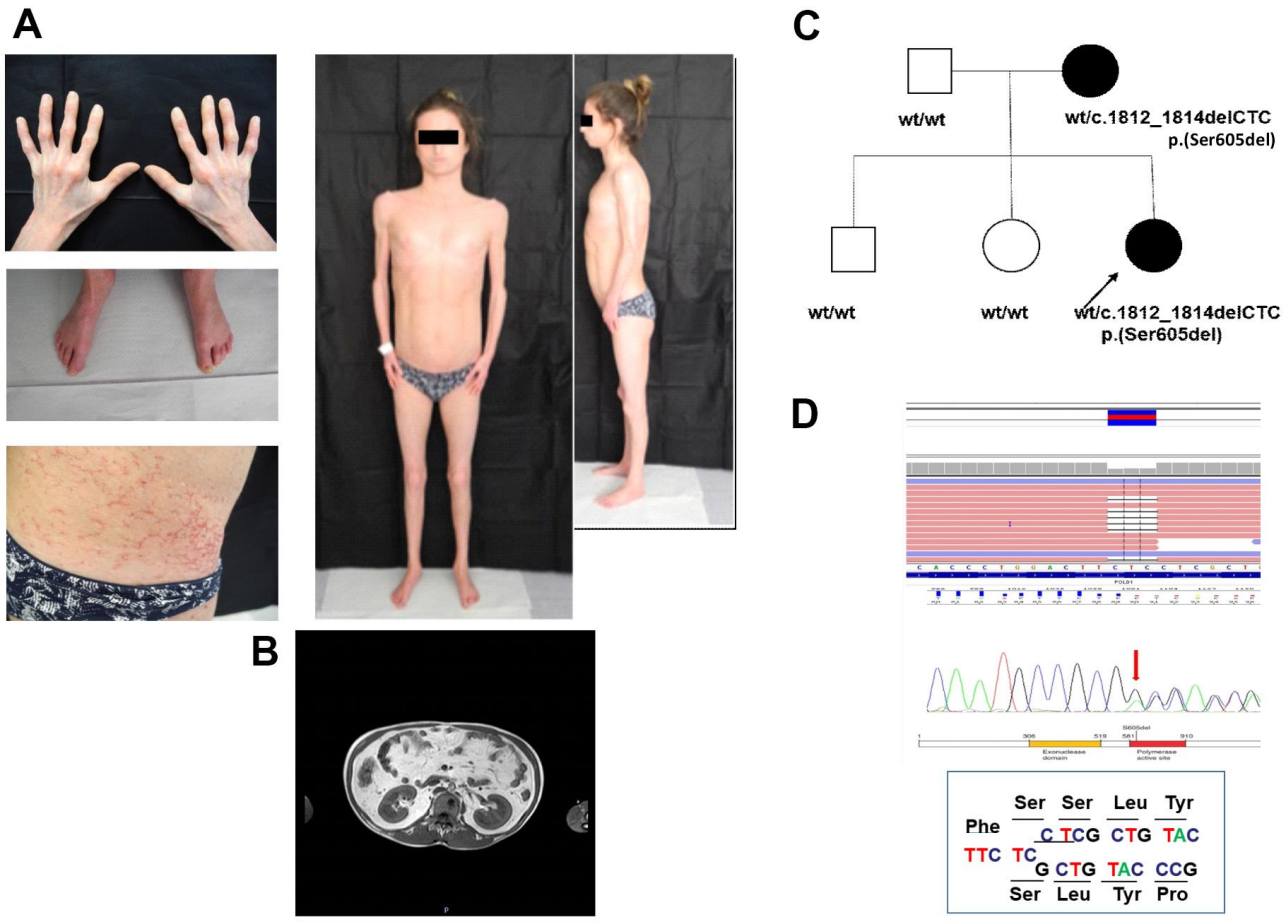
**Clinical and molecular diagnosis.** (**A**) Pictures showing patient’s clinical features at the age of 21: telangiectasia and skin scleroderma, minimal subcutaneous adipose tissue and reduced limb muscle mass. (**B**) MRI of the abdomen shows an over-representation of mesenteric fat. (**C**) Proband’ family pedigree. Full symbol indicates MDPL affected family members. (**D**) DNA analysis of the proband by NGS and Sanger sequencing revealed the recurrent heterozygous single amino acid in frame genetic deletion c.1812_1814delCTC, p.Ser605del in *POLD1* gene, segregating as an autosomal dominant variant.

MDPL clinical diagnosis was done at 21 years old, although the mother reported growth retardation since the proband was 3 years old (Figure 1A). At the time of the diagnosis, the patient was normotensive with a BMI of 19.5. Although fasting plasma glucose concentration was normal, an oral glucose challenge revealed type 2 diabetes with marked hyperinsulinemia compatible with extreme insulin resistance. Skin scleroderma, telangiectasia, and subcutaneous lipoatrophy were clinically evident throughout the body concurrently with reduced limb muscle mass (Figure 1A). A DEXA scan detected percentages of fat at both the upper and lower limb <10%, whereas bone mineral density at lumbar and femoral sites was in the osteopenic range (data not shown). MRI of the abdomen showed an over-representation of mesenteric fat, as reported in other cases of MDPL (Figure 1B). Finally she had primary amenorrhea. Clinical signs of MDPL in the mother were very mild.

### MDPL syndrome: a case of germline *POLD1* variant

DNA sequencing of the proband by Next Generation Sequencing (NGS) reveals the heterozygous deletion c.1812_1814delCTC, p.Ser605del in *POLD1* gene, segregating from the mother (Figure 1C, 1D), while healthy siblings and father are negative. The mother shows a mosaic pattern of the MDPL-causing variant in peripheral lymphocytes, hair, urine and saliva (20%, data not shown).

### Nuclear envelope characterization and micronuclei assessment of MDPL-HDFs

Once established primary cell lines from dermal fibroblasts (HDFs), cells were first analysed for nuclear architecture instability, a sign that usually correlates with progeroid disorders, especially those related to alterations in nuclear lamina [17]. For each comparison among MDPL-HDFs and WT-HDFs we used age-matched cells at the same passage doubling. Several defects of nuclear shape were identified in MDPL-HDFs including invaginations, large protrusions (blebs) and doughnut-shaped nuclei, with a frequency higher than in WT-HDFs (7.3% respect to 1.5%; *P*<0.01) (Supplementary Figure 1A–1C). In order to analyze the nuclear envelope proteins, immunofluorescence analyses were conducted both for the detection of prelamin and mature lamin A. Prelamin A showed an abnormal accumulation in about 30% of MDPL nuclei, mostly located at the nuclear rim, within membrane invaginations and occasionally in intranuclear structures. As expected, prelamin A was rarely detected (4,3%) in WT cells which did not show any nuclear alterations (Supplementary Figure 1A, 1D; *P*<0.001). Meanwhile, lamin A was expressed in all nuclei both in WT and MDPL-HDFs with the same nuclear rim distribution (Supplementary Figure 1B).

Moreover, nuclear immunolabeling revealed the presence of micronuclei in quantity about 4 times more in MDPL cells (2.85%) than in WT ones (0.7%) (Supplementary Figure 2A; *P*<0.01), as previously described by us on another MDPL patient [18]. Thus, in order to acquire a broad vision about MDPL micronuclei transcriptional and replicative potential, we also performed a further immunofluorescence analysis through the immunostaining with anti-lamin B1 and anti-histone H3 antibodies (Supplementary Figure 2B, 2C). More than 70% of MDPL micronuclei resulted to be positive both for lamin B1 and H3 (Supplementary Figure 2B, 2C), while all WT micronuclei were negative for lamin B1, and only 10% of them resulted to be positive to H3 immunostaining (data not shown).

**Figure 2:**
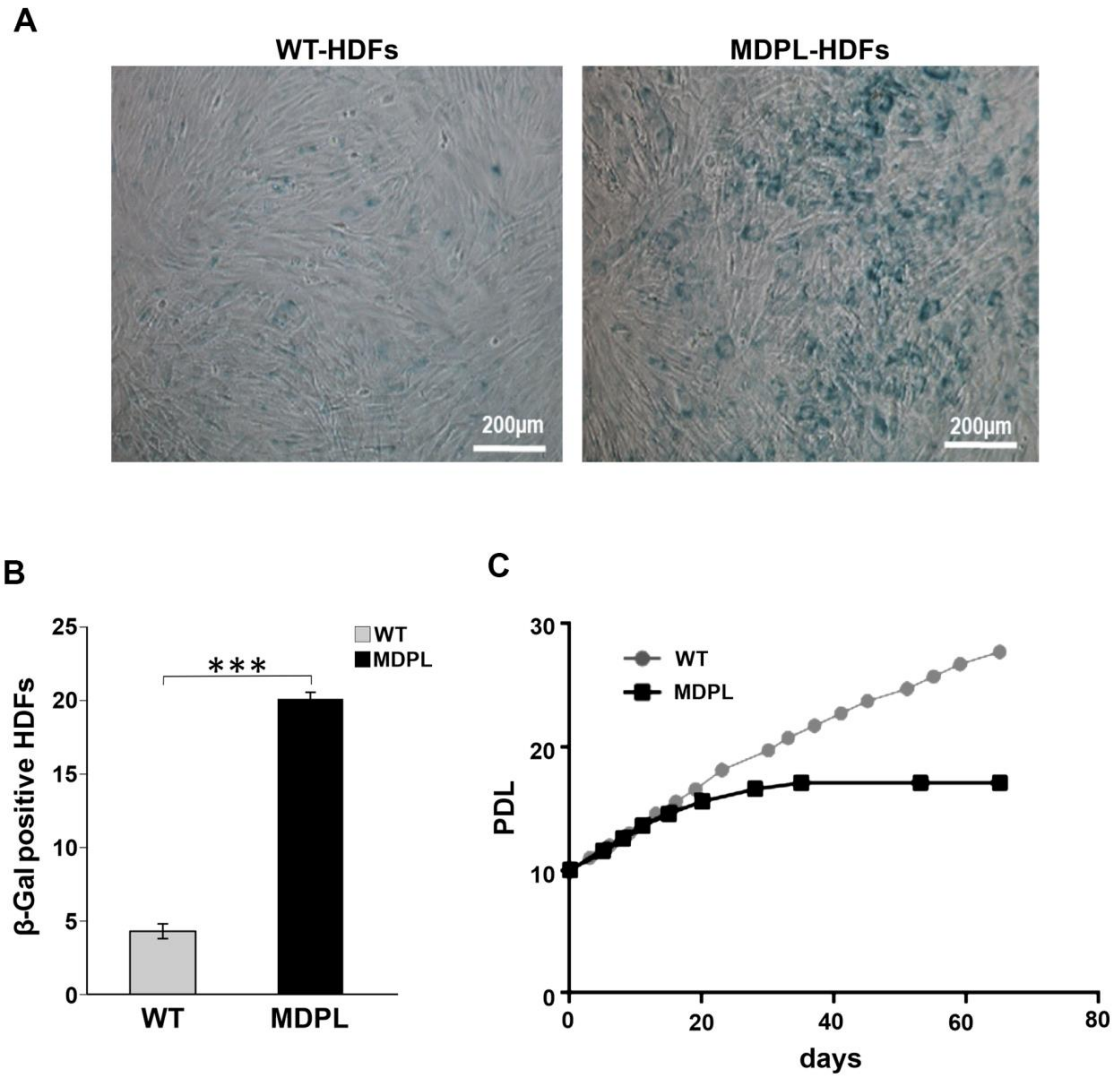
**Senescence-associated β-galactosidase assay in MDPL-HDFs and WT controls.** (**A**) Representative image of Senescence-associated β-galactosidase assay. A greater amount of intensely positive blue cells are displayed in MDPL-HDFs than in WT controls. (**B**) The histogram shows the average percentage of β-galactosidase-positive cells in WT (4,3%) and MDPL fibroblasts (20%). Error bars represent the SD from the analysis of 100 cells from three independent experiments and WT values are displayed as the average percentages of 2 different controls (***P < 0.001). (**C**) Long-term culture of WT (grey) and MDPL HDFs (black). PDL: population doubling levels.

### Cellular senescence and growth trend of MDPL-HDFs

To further explore the leading senescence process, a β-galactosidase assay has been performed in which WT and MDPL-HDFs have been compared at the same number of passages. A higher number of senescent cells was detected in MDPL-HDFs (20%) when compared to WT β-galactosidase-positive cells (4.3%) (p<0.001) (Figure 2A, 2B). Successively, we evaluated a possible growth decline along a long-term culture, exploring growth trend in relation to the length of the doubling time. MDPL-HDFs showed a growth decrease parallel to an increase of the doubling time, leading to a growth crisis with clear signs of cellular senescence (Figure 2C).

### DNA repair process and Polδ expression in MDPL-HDFs at basal level and after cisplatin treatment

**Figure 3:**
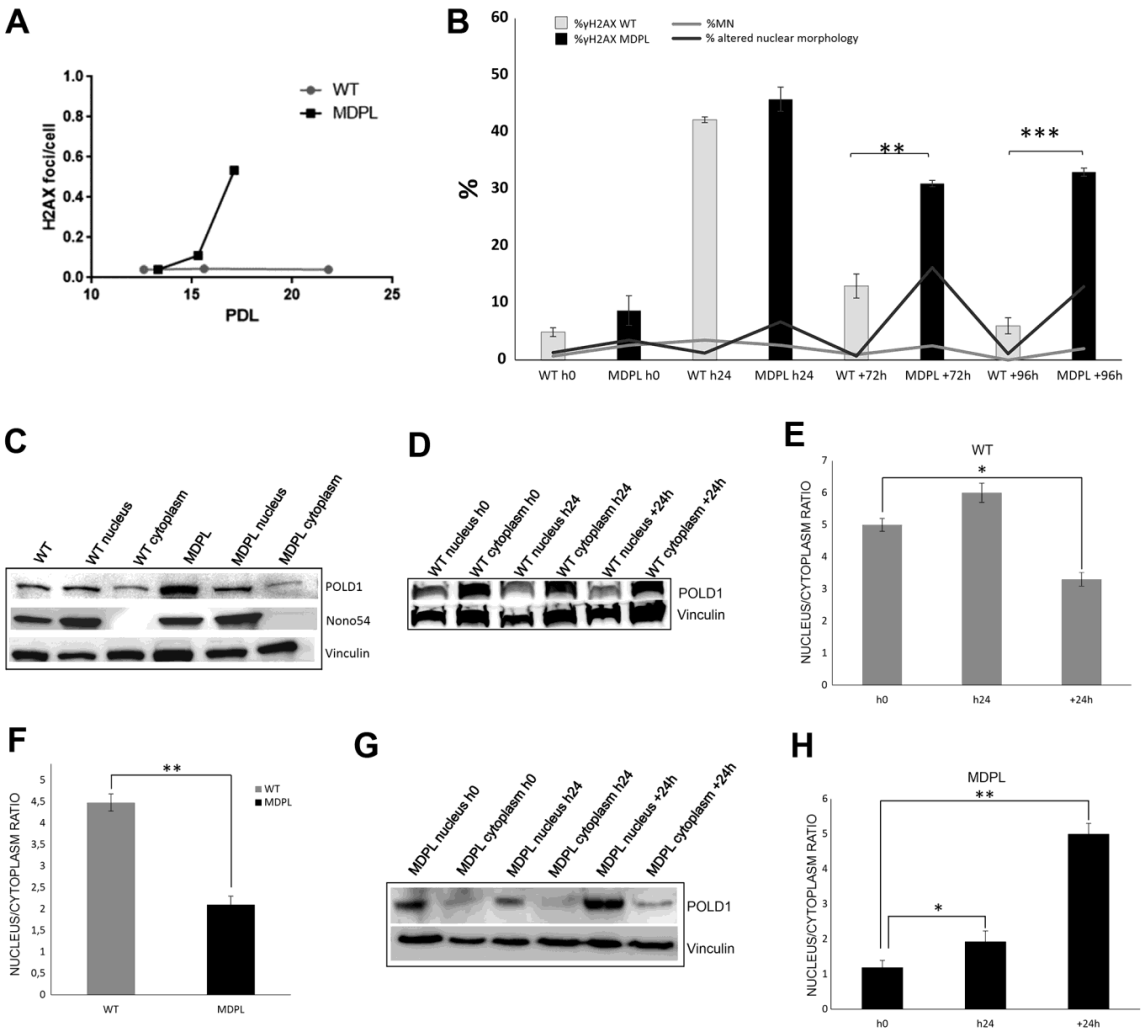
**DNA repair and protein expression after cisplatin treatment.** (**A**) γH2AX foci in fibroblasts at different population doubling levels (PDL). (**B**) The graph shows the trend of γH2A.X positive cells at each time point. Error bars represent the SD from the analysis of 100 cells from three independent experiments and WT values are displayed as the average percentages of 2 different controls. (**P<0.01, ***P< 0.001). The two lines show the trend of micronuclei (light grey line) and altered nuclear morphologies (dark grey line) in MDPL-HDFs compared to WT cells, at each time point after cisplatin treatment. The percentage of micronuclei is around 2.5% in MDPL-HDFs+72h, while it decreases to ~1% in WT cells+72h (*P<0.05). After further 24h (WT and MDPL-HDFs +96h) the percentage of MN remains around 2% in MDPL-HDFs, decreasing to 0% in WT-cells (*P<0.05). Also the difference between the percentage of altered nuclear morphology is statistically significant between WT and MDPL-HDFs both at +72h and +96h (**P<0,01); (**C**) Western blot analysis of Polδ from MDPL and WT HDFs and following nuclear cytoplasm fractionation. Nono54 was used to check the correct fractionation and Vinculin was used as control. (**F**) Densitometric Analysis of Polδ nucleus/cytoplasm ratio protein levels. (**P<0.01). Western blot analysis of equal amount of total proteins from WT (**D**, **E**) and MDPL-HDFs (**G**, **H**) at h0, h24 and +24h of cisplatin treatment and following nuclear cytoplasm fractionation. (*P<0.05, **P<0.01). Vinculin was used as control. Data are presented as means ± SD.

In view of the possible connection between the observed MDPL cellular progeroid phenotype and DNA damage accumulation, we have successively evaluated the number of DNA double-strand breaks (DSBs) rejoining by measuring phosphorylated H2AX (γH2AX) foci per cell at different population doubling level (PDL). γH2AX foci are considered as a valuable marker of DNA double strand damage: while this number remained constant in WT-HDFs, there was an early increase (2.5 fold) in MDPL-HDFs, followed by a marked (13-fold) rise at senescence stage (Figure 3A).

This data was also evaluated after cisplatin-induced damage, along the kinetics of DNA repair. Positive cells were counted after 72 and 96 hours from cisplatin treatment (Figure 3B, Supplementary Figure 3A).

About 50% of cells, both WT and MDPL, showed γH2A.X foci right after the end of the treatment (WTh24 and MDPLh24). Then the amount of foci-positive HDFs starts decreasing in both cells, even if it remains significantly higher in MDPL-HDFs than in WT ones. In fact, after 72 hrs from the end of cisplatin exposure, a significant reduction of γH2A.X signal was observed only in WT cells (WT+72h: 13%), while the percentage continued to be statistically higher in MDPL-HDFs (MDPL+72h: 31%) (P<0.01). Interestingly, after 96 hrs from the end of cisplatin exposure, the 33% of residual phospho-H2A.X positive MDPL cells were observed (MDPL+96h), compared to 6% of residual positive WT cells (WT+96h) (*P*<0.001) (Figure 3B). The immunofluorescence analysis of γH2A.X signal was also quantified evaluating the fluorescence intensity, indicated as high expression (+++ and ++++) of DNA damage response marker in MDPL nuclei for each experimental condition. This data also confirmed the persistence of unrepaired DNA DSBs at later time points post damage (Supplementary Figure 3B).

In addition, to the counting γH2A.X foci-positive cells, we have evaluated the recovery of the induced damage by quantifying the altered nuclear morphology and the presence of micronuclei in cisplatin-treated HDFs over the time. The rescue showed a statistically significant trend, because MDPL-HDFs recovered more slowly than WT ones and the recovery is never complete, as indicated by the persistent presence of nuclear alterations and micronuclei even 72 and 96 hours after cisplatin treatment (Figure 3B). The percentage of micronuclei remains around 2% in MDPL-HDFs while decreases to 0 in WT cells at 96hours (*P* < 0.05). Nuclear alterations show the same trend, being about 13% in MDPL-HDFs and decreasing to 1% in WT ones *(P* < 0.01).

Polδ expression has been evaluated both at basal level and after cisplatin treatment. We compared MDPL-HDFs versus WT ones by Western blotting analysis, after a nuclear fractionation experiment for separating the nucleoplasmic fraction from the cytoplasmic one. The correct fractionation was checked by using a nuclear marker (nono54) (Figure 3C). At basal level, the nuclear to cytoplasmic *ratio* of Polδ protein shows a strong decrease in MDPL cells, if compared to WT ones (*P*<0.01) (Figure 3C, 3F). In Figure 3D the same protein analysis was done after cisplatin treatment. Right after the drug exposure (h24), Polδ nuclear to cytoplasmic *ratio* of WT cells resulted to be slightly increased than the basal condition, before declining under the basal value after 24 hours (+24h) from drug removal (Figure 3D, 3E; P<0.05). This trend varied in a different manner in MDPL-cells where Polδ *ratio* showed a progressive increase both immediately after cisplatin treatment (h24, P<0.01) and after 24 hours (+24h) from drug removal (Figure 3G, 3H; P<0.01).

### FACS analysis of cell cycle after cisplatin treatment

**Figure 4:**
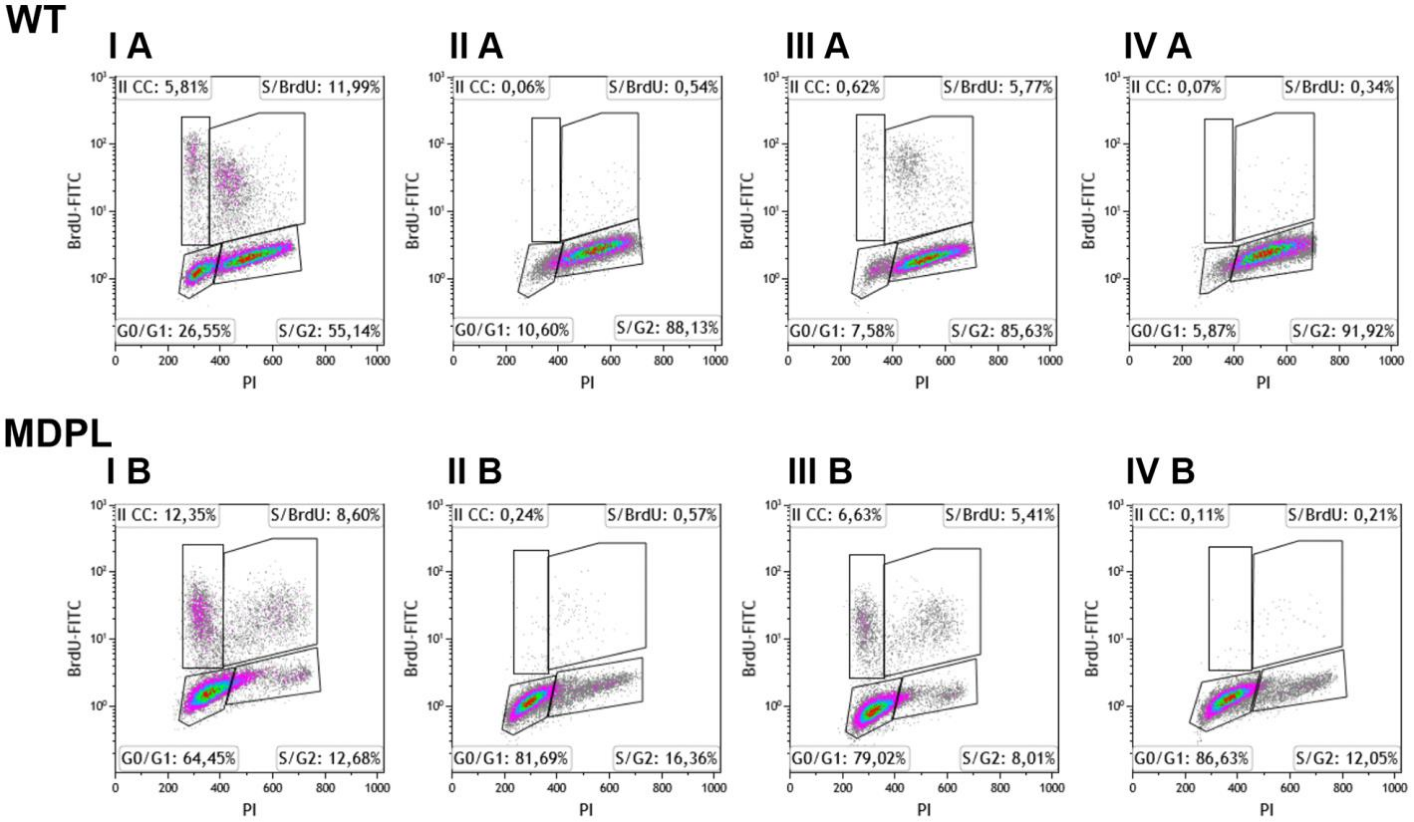
**Flow cytometry analysis of BrdU-positive cells of WT and MDPL HDFs.** Representative histogram of cell cycle profiling, reporting the insets with the relative percentage of cells in different phases of cell cycle (G0/G1, S and G2/M). (**I A**, **I B**) indicate 48 hours of cell culture + 6 hours of BrdU. (**III A**, **III B**) indicate 72 hours of cell culture + 6 hours of BrdU. (**II A**, **II B**) indicate 24hours of cell culture + 24 hours of cisplatin treatment. (**IV A**, **IV B**) indicate drug effect after 24 hours from cisplatin removal.

To further explore and compare the distribution profiles between the MDPL and WT cells in the various phases of the cell cycle (G0/G1, S and G2), flow cytometric analysis was performed after BrdU incubation. Results are shown in Figure 4. WT HDFs enter (about 18%) in cell cycle with a subpopulation of proliferating cells (~ 6%) and are also able to re-enter in a second cell cycle (II CC). More than 80% of cells are not cycling cells but they remained distributed in G0/G1 (~ 26%) and S/G2 (~ 55%) (IA).

In the same culture condition, after 6h of BrdU incubation, more than 20% of MDPL-HDFs was cycling and a high percent of these cells (~12%) enter in a second cycle. Differently from WT ones, more than 77% of cells are no non-cycling cells that remained blocked in G0/G1 phase (~ 64%, IB).

Similar distribution is observed after 72h of cell culture, nevertheless a reduced amount of cells was cycling, and ~6% (IIIA) and ~ 12% (IIIB) of WT and MDPL cells respect to the situation registered at 48h (IA and IB). Noteworthy, the number of MDPL cycling cells was twice respect to the control cells and also a high percentage of them are entering in a second cycle (~6.5% vs 0.6%). Again, more than 90% of the WT cells remained distributes in G0/G1 (~7%) and S/G2 (~ 86%) (IIIA) while more than 85% of the MDPL cells remained in G0/G1 (~ 79%) and the residual in S/G2 (~ 8%) (IIIB).

The cisplatin treatment for 24h determined a complete blockade of proliferation evaluated at both 24 (IIA, B) and +24 hours after the drug removal (IVA, B) with a not significant recovery of the proliferation capability of both cells. Consistently with the untreated cell cultures, the non-cycling WT cells remained distributes in G0/G1 (~ 11%) and S/G2 (~ 88%) (IIA), while ~ 81.69% of non-cycling MDPL cells remained blocked in G0/G1 phase (IIB). Overlapping results were obtained analyzing both cells at +24h, namely after the drug removal (IVA, IVB).

### Evaluation of Polδ expression, genomic and telomeric damage and repair after 1 Gy X-irradiation

**Figure 5:**
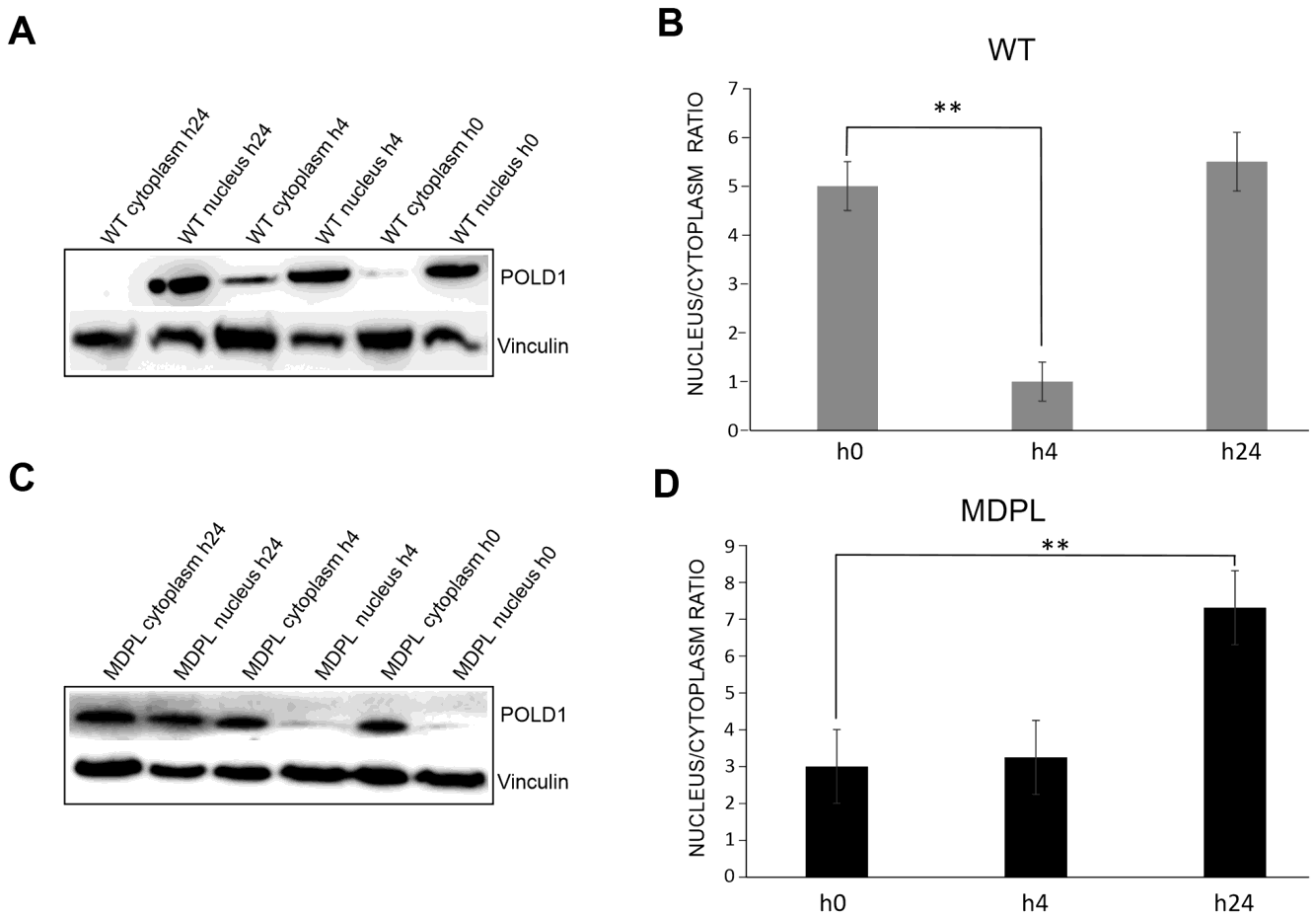
**Western blot analysis of Polδ from MDPL and WT HDFs after 1 Gy-X-irradiation.** (**A**) Western blot and (**B**) densitometric analysis of Polδ nucleus/cytoplasm ratio protein levels after 1 Gy-X-irradiation in WT-HDFs. (**P<0.01). (**C**) Western blot and (**D**) densitometric analysis of Polδ nucleus/cytoplasm ratio protein levels after 1 Gy-X-irradiation in MDPL-HDFs. (**P<0.01). Data are presented as means ± SD.

**Figure 6:**
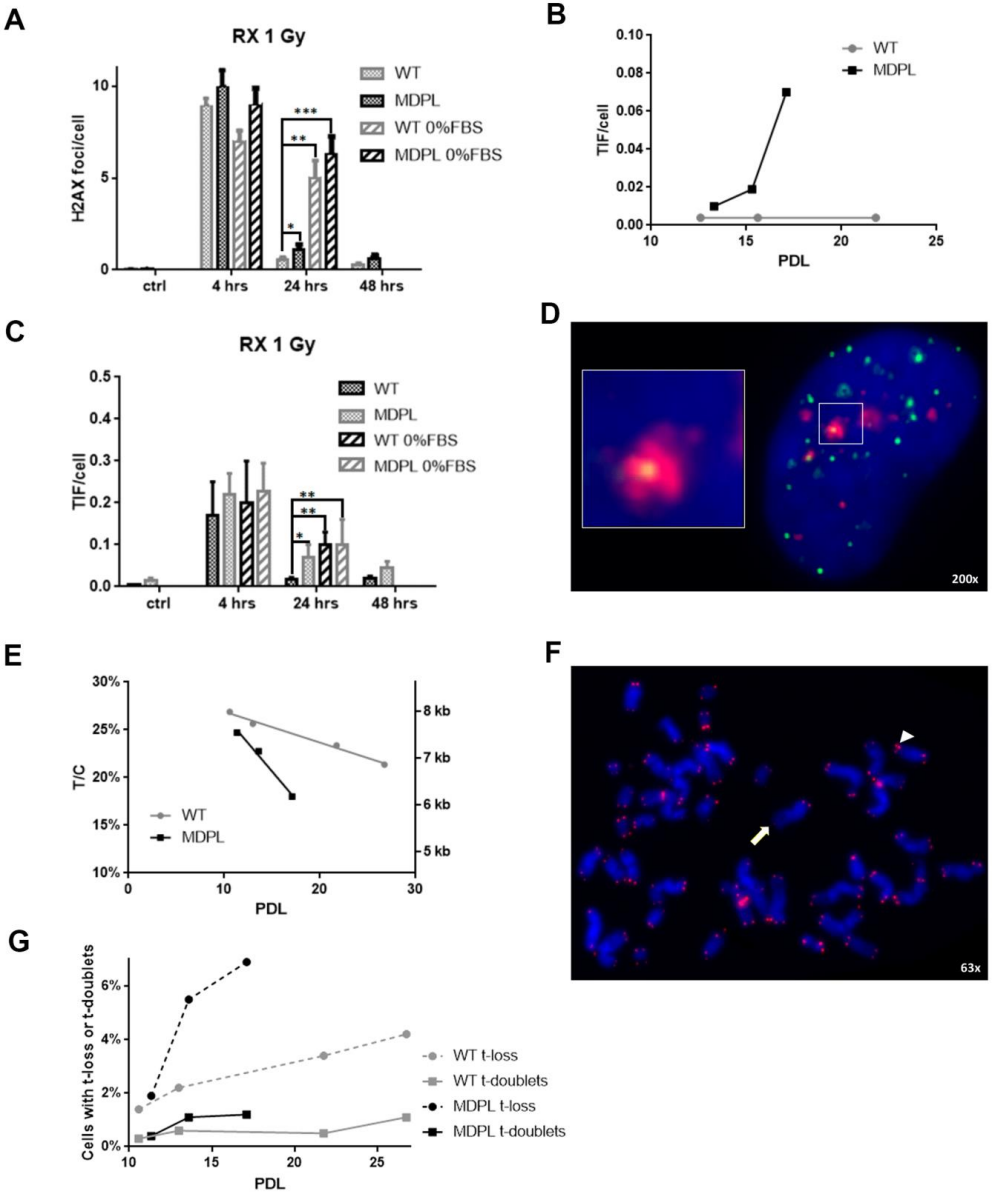
**DNA repair kinetics after 1 Gy of X-irradiation.** (**A**) DNA repair kinetics evidenced by γH2AX foci in serum-fed and serum-depleted cells after 1 Gy of X-irradiation. (**B**) Telomere-induced foci (TIF) in unirradiated fibroblasts at different population doubling levels (PDL). (**C**) Time course of TIF in serum-fed and serum-depleted cells after 1 Gy of X-irradiation. (**D**) Representative image of Telomeres stained with anti-TRF1 antibody (green), foci stained with anti-γH2AX antibody (red), and DAPI-stained nucleus (blue). Magnification 200X; inset shows co-localization of both antibodies, indicating a TIF. (**E**) Ratio between telomeric and centromeric fluorescence: T/C. (**F**) Representative image of chromosome spread showing telomere doublet (arrowhead) and telomere loss (arrow). Magnification 63X. (**G**) Telomere loss (circles, dashed lines) and telomere doublets (squares, continuous lines) at different PDL. WT in grey, MDPL in black.

We firstly evaluated the effect of 1 Gy X-irradiation damage on the expression of Polδ by Western Blot analysis performed on WT and MDPL HDFs. The nuclear to cytoplasmic ratio in WT HDFs decreases 4 hour after irradiation (P<0.01), but returns comparable to the basal condition 24 hours after the irradiation (Figure 5A, 5B). In MDPL HDFs the ratio value remains unmutated after 4h but interestingly strongly increase 24 hours from the time of damage induction, revealing a boost of the nuclear component (Figure 5C, 5D; P<0.01). Successively, also the kinetics of DNA repair was studied evaluating the presence of γH2AX foci. The experiments were performed on low passage fibroblasts (12-13 population doublings), when rates of growth were identical and no signs of aging were present. The number of foci was counted 4, 24 and 48 hrs after irradiation. Four hours after irradiation, the level of residual γH2AX foci was not significantly different between WT and MDPL-HDFs (Figure 6A). On the contrary, after 24 hours, γH2AX foci per cell in MDPL-HDFs were twice the ones in WT-HDFs (1.1 *vs* 0.53, *P*=0.041). The same condition was registered after 48 hours, although it was not statistical significant (*P*=0.052). In order to study DNA repair in cells that do not pass through the S phase, we repeated the same experiment on serum-depleted fibroblasts (Figure 6A; WT 0%FBS and MDPL 0%FBS). Comparing γH2AX levels in serum-fed versus serum-depleted cells after 24 hours from irradiation, the latter showed a 5-6 times higher value (*P*=0.0015 and *P*=0.0006, respectively). Thus, we decided to evaluate the baseline levels of those γH2AX foci localised at telomeres (telomere induced foci, TIF) in both cell lines at different population doubling levels (Figure 6B). MDPL HDFs clearly showed more TIF per cell even at low PDL (0.01 in MDPL vs 0.004 in WT). Again, there was an increase at mid-time, followed by a 17-fold increase at senescence. On the contrary the levels of TIF in WT HDFs remained constant through the duration of the whole experiment. Soon after, on the same slides with irradiated fibroblasts, TIF were analysed (Figure 6C, 6D). The results were almost the same as those for γH2AX foci. Serum-fed and serum-depleted cells had almost the same levels of TIF after 4 hours. Nonetheless, the difference between WT and MDPL HDFs 24 hours after irradiation was even more dramatic (0.018 vs. 0.07, *P*=0.041). TIF levels in serum-depleted fibroblasts, both WT and MDPL, were identical between them and also similar to serum-fed MDPL cells (Figure 6C).

### Telomere shortening

Successively also the telomere lengths at different PDL were evaluated and shown in Figure 6E, 6F. Besides relative values expressed as the ratio between telomeric and centromeric fluorescence (T/C), we also converted these values in Kb, following the formula of Perner et al., [19] calibrated with tumor cell lines in our laboratory (Supplementary Figure 4). The rate of telomere shortening was greater in MDPL HDFs compared to the WT ones, approximating 0.24 Kb vs. 0.066 Kb per PDL.

On the same chromosome spreads where we measured telomere lengths, we also took note of those chromosome arms that showed no telomeric signal (telomere loss) or disrupted signals (telomere doublets), strongly considered as marker of telomeric replication stress (Figure 6F, 6G). Figure 6G clearly shows that telomere loss paralleled telomere shortening and telomere doublets showed an earlier increase in MDPL HDF.

## DISCUSSION

Mandibular hypoplasia, deafness, and progeroid features, with concomitant lipodystrophy define the MDPL syndrome, a very rare disease displaying a spectrum of clinical characteristics with variable expression in the developmental stage of the phenotype. MDPL and Mandibuloacral Dysplasia type A (MADA; OMIM #248370) or Hutchinson-Gilford progeria syndrome (HGPS; OMIM #176670) show overlapping clinical manifestations and belong to similar groups of syndromes characterized by premature aging and inheritable nuclear envelope and/or DNA repair defects.

To date, only 26 patients with MDPL syndrome have been reported. Among them, 20 patients had the recurrent in frame deletion of a single codon (c.1812_1814delCTC, p.Ser605del), including our previous case reports [18, 20] with clearly established pathogenicity. Three patients had the missense variant (c.1572C>T, p.Arg507Cys) affecting the polymerase domain and the C-terminal part of the proofreading (exonuclease) domain of the polymerase [20, 21]. Only one presents a novel variant named c.3209T>A, p.Ile1070Asn in the Zinc Finger 2 domain of the protein. In the same protein domain, a novel inherited *POLD1* missense variant (c.3199 G>A; p.Glu1067Lys) has been recently identified in two related female patients with milder signs and classified as likely pathogenic [22].

Human DNA Polδ is a multi-subunit complex, highly conserved and ubiquitously expressed, composed of four elements: p125, p68, p50 and p12 [4]. p125 has been identified as a catalytic subunit and is encoded by the *POLD1* gene in human. When DNA damage occurs, Polδ together with p50 e p68 subunits is rapidly recruited into the nucleus to the damaged DNA, which allows the assembly of protein complexes involved in DNA repair.

Previously, we reported two MDPL patients [23, 18] carrying p.Ser605del heterozygous deletion in *POLD1* gene. Functional studies have shown that the deletion results in an enzyme which is inactive as a polymerase, but retains the exonuclease activity. In particular, S605 is located in the highly conserved motif A of the polymerase active site, that was shown to be able to bind DNA, but not to catalyse polymerization. Hence, it is speculated that germline mutations in the active site of the polymerase might increase replication stress, resulting in a progeroid disorder [23]. In patient’s fibroblasts, we observed severe nuclear envelope abnormalities, presence of micronuclei, accumulation of prelamin A, altered cell growth, cellular senescence and a delayed DNA damage response after cisplatin exposure. These data provided the first suggestion of an altered genome maintenance contributing to MDPL condition.

In this study, we describe the *in vitro* characterisation of the recurrent in frame deletion (c.1812_1814delCTC, p.Ser605del), within *POLD1* gene in a 21 years old female’s primary dermal fibroblasts. We confirmed marked nuclear envelope abnormalities, presence of micronuclei, an abnormal prelamin A accumulation and cellular senescence, which represent typical cellular features of progeroid syndromes, such as MADA and HGPS. Several lines of evidence have linked nuclear defects, such as micronuclei and nuclear buds, with increased genomic instability, DNA repair defects and anomalies in the nuclear lamina. In fact, MDPL cells are characterized by micronuclei presence at basal condition in about four folds greater than WT ones. The presence or absence of lamin proteins around micronuclei has important implications for the phenotypes of cells, because it correlates with transcription or replication process inside micronuclei. Most chromosome-type lamin B-positive micronuclei are generated from the anaphase laggards, while most negative micronuclei from the anaphase chromatin bridge [24]. Genes in lamin B-negative micronuclei are not expressed (in our case 28.57%), whereas genes in lamin B-positive micronuclei (in our case 71.4%) are actively expressed and might cause irregularities that affect cell phenotypes. Further characterization was performed with Histone H3 antibody in order to define transcriptionally active regions of the genome and in MDPL-HDFs only 22% of micronuclei was H3 negative by immunofluorescence analysis.

Furthermore, MDPL-HDFs growth rate decreased earlier leading to a growth crisis with clear signs of cellular senescence. This feature is clearly underlined by cell proliferation and β-galactosidase assays and further confirmed by FACS analyses that revealed an arrest of MDPL-HDFs in G0/G1 phase transition with a lower number of cells in the S phase, while the WT counterpart mostly accumulates in S phase. In details the dynamic cell cycle analysis suggests that the variant in *POLD1* gene determines a marked cell cycle blockage in G0/G1 phase, noticeable in all the conditions investigated. These results are to some extent in accordance with those reported by Zeng et al [8] and Song et al [9], who emphasize the role of *POLD1* in the regulation of cell cycle progression. Nevertheless, the higher percentage of MDPL cells respect to WT ones that enter in cell cycle, as demonstrated by increased incorporation of BrdU, do not agree with the results of culture proliferation rate (Figure 3). A possible explanation could be represented by increase propensity of MDPL cells to trigger programmed cell death during the cell cycle. Anyway, the results obtained from the proliferation assays and cells cycle analyses are sufficiently clear to support the hypothesis of a defect in the control of DNA replication process by the in frame deletion in *POLD1* gene.

These results confirm those already described in our previous work [18] and support the possibly shared pathophysiological mechanisms among *POLD1*, *LMNA*-related progeroid diseases and lypodystrophic syndromes. The DNA Polδ is fundamental in genome maintenance and it is involved in DNA repair processes. The outcomes acquired labelling cells with anti-γH2AX antibody support the hypothesis concerning the impaired ability of DNA damage repair in MDPL cells. Specifically, while the baseline levels of γH2AX foci positive nuclei remain constant in WT-HDFs, a marked enhancement is assessed in MDPL-HDFs at increasing PDL. The diminished DNA repair ability is even more marked after cisplatin-induced damage. In fact, cisplatin-induced intrastrand adducts are bulky lesions that interfere with DNA replication machinery. The prolonged stalling of replication forks can result in the formation of DNA DSBs, and such deleterious damage can lead to gross DNA rearrangements or cell death [25]. If an impaired repair or an excessive damage occurs, cells undergo apoptosis [26]. After damage, in WT cells Polδ is recruited to the nucleus and then its amount decreases after 48 hours, as demonstrated also by the reduction of γH2AX foci. In MDPL-HDFs the situation is more intricate, because the pathogenic variant leads to an enhancement of nuclear Polδ amount, even if the mutated protein is not able to carry out its function. This situation results in an increment of γH2AX foci and a constant increase of nuclear protein amount, likely due to an attempt to reduce DNA damage. This inability to correct the induced damage is also evidenced by the persistence of micronuclei and of nuclear alterations at later time points post damage.

Successively another type of damage was also induced, X 1Gy irradiation, because it allows to study the kinetics of DNA repair alone, differently from chemical treatment, where induction of damage and its repair coexist. In the kinetics of double strand breaks (DSB) repair, there is a fast component repairing around 80% of the initial damage during the first 4 hours, during which Non-Homologous End-Joining (NHEJ) mainly operates, and a late component, when Homologous Recombination (HR) seems prevalent [27]. The reduced repair ability of MDPL-HDFs is clearly linked to HR, as showed in experiments performed with serum-depleted cells (0% FBS), in which the process of HR is almost abrogated. In this condition in fact there are no differences between mutated and wild-type fibroblasts. This should be not unexpected, since Polδ (the polymerase of which p125 is a subunit) is a component of the HR machinery [28].

Considering that DNA repair by NHEJ at telomeres is precluded by the shelterin complex, whereas this task can be performed by HR [29], we studied the DNA damage induced at the end of chromosomes. Our results indicated, in fact, that the reduced efficiency of Polδ in MDPL-HDFs affects, in particular, DNA damage induced at telomeric sites, confirming that the main mechanism that can repair telomeric DSB is compromised. To confirm this hypothesis are the results that X-ray-induced TIF in MDPL-HDFs are the same as in serum-depleted wild-type fibroblasts (in which HR is almost abrogated). In this case, while in WT-HDFs nucleus Pold remains comparable to the basal condition after 24 hours of damage, in MDPL-HDFs there is a continuous boost in protein expression in the nucleus, recruited to repair DNA damage. Beside the consequences after a serious injury such as X-irradiation, the reduced efficiency of Polδ exerted its effects also on the lifespan of untreated MDPL-HDFs. Since human fibroblasts have no telomerase activity, their telomere length cannot be restored after it has been shortened [30]. As unrepaired telomeric damage leads to telomere shortening, our data indicated that MDPL-HDFs, suffering more damage at their telomeres (as shown by TIF, but also by telomere doublets), undergo a faster rate of telomere shortening, leading to an earlier onset of cellular senescence.

The impressive clinical resemblance among LMNA-linked progeria and MDPL syndrome is accompanied by common pathophysiological pathways. In this study, we demonstrated several hallmarks of aged cells in MDPL-HDFs, including genetic damage, telomere shortening, cell senescence and defect of proliferation. Additional experiments aiming to focus on protein partners of Polδ can be useful to expand the scenario of the persistence of unresolved repair foci and telomere shortening in progeroid laminopathies due to the altered lamin network. On the other hand, molecular links identified in premature aging conditions could be verified in MDPL cells.

The altered and shortened structure of the chromosomes surely impairs their interaction with the inner membrane proteins and, together with the unusual presence of prelamin, leads to a modification of the cellular fate. It is known that the increase in prelamin causes ROS boost and oxidative stress that in turns induce post-translational modifications of the inner nuclear membrane emerin [31]. Our preliminary data suggests an altered mitochondrial activity and unbalanced autophagy probably caused by an impaired DNA repair and genomic instability (data not shown). This interesting aspect is going to be further developed. Moreover telomere dysfunction in HGPS cells induces the transcription of telomeric non coding RNA (tncRNA) which usually control the DNA damage response, increasing cellular senescence [32].

Finally, altogether our results suggest that alterations in DNA replication/repair function of *POLD1* could be considered as a primary causes of molecular pathogenesis of this rare disease. The knowledge of the link between *POLD1* defects and the development of highly diverse clinical symptoms ranging from depletion of subcutaneous fat to deafness and bone abnormalities strongly represents a key challenge.

In order to respond to some of these intriguing questions, we are now generating human induced pluripotent stem cells from patient’s fibroblasts for applying genome-editing technologies, like the CRISPR-Cas9 nucleases, as tool for drug screening applications targeting DNA repair and development of therapeutic treatment.

## MATERIALS AND METHODS

### Clinical phenotype and genetic analysis

The proband at 21 years old is characterized by a complex clinical phenotype with loss of subcutaneous fat, a typical facial appearance with mandibular hypoplasia, prominent eyes and nose. These features are associated with sensorineural deafness and metabolic abnormalities including insulin resistance and diabetes. After informed consent, blood sample was obtained and genomic DNA was extracted using EZ1 DNA Blood 200μl purification kit (Qiagen, GmbH, Germany). NGS was performed using a custom-built NGS panel which includes some of the main genes related to progeroid syndromes (*LMNA*, *ZMPSTE24, WRN, POLD1, BANF1*). We used two specific primers pool to cover the 98.9% of the entire coding sequence of all genes analysed. The sequence analysis was performed using the Ion S5 platform (Thermo Fisher Scientific). Run analysis was performed using Integrative Genomics Viewer (IGV, Broad Institute) (http://www.broadinstitute.org/igv/). The validation and the successively segregation analyses of the variants identified have been performed through Sanger sequencing, using ABI 3130 XL Genetic Analyzer (Life Technologies) using the BigDye Terminator v3.1 Sequencing Kit (Applied Biosystems). We used the reference sequence of *POLD1* gene by ENSEMBL (http://www.ensembl.org/index.html).

Blood, and saliva were obtained from all the family members to further investigate the segregation of the variant and the percentage of mosaicism.

### Cell cultures

Skin punch biopsy was obtained from patient and two healthy donor using standard procedure (e.g. obtained with 4mm round Visipunch instrument) and kept in complete DMEM 20% FBS media on ice. Immediately after collection, the sample was rendered sterile by 3 consecutive washes in PBS (DPBS-Dulbecco's Phosphate-Buffered Saline; w/o calcium, w/o magnesium; Thermo Fisher Scientific) and antibiotic-antifungal (PAA, The Cell Culture Company), then it was placed in a solution of Dispase (2mg/mL; Gibco) overnight at 4° C, in order to cleave the components of the extracellular matrix. The following day, using a pointed forceps, the epidermis was separated from the dermis and it was dissected into 12-15 evenly sized pieces using a scalpel. Pieces were incubated with Collagenase I for 4h at 37° C and then transferred into Tissue Culture Plates pre-treated with gelatin, in DMEM High Glucose (Gibco) media, containing 10% FBS (Gibco), 1% L-Glutamine (PAA, The Cell Culture Company), 1% Penicillin/Streptomycin (PAA, The Cell Culture Company), 1% NEAA (Gibco) and 0.1% β-mercaptoethanol (Gibco). After about 25 days, the outgrowth of primary fibroblast (HDFs) was observed, expanded and then analyzed. Skin biopsies were obtained from patient and donors according to The Committees on Health Research Ethics of Tor Vergata Hospital (2932/2017) and EU ethical rules.

All the *in vitro* experiments were performed comparing cells (proband and two control wild type samples) at same age, sex and cell doublings WB analyses were performed using only one WT control.

### Senescence-associated β–galactosidase staining

SA-β-gal activity assay was performed according to the manufacturer’s protocol (BioVision Senescence detection kit, K320-250). Briefly, HDFs (p10) were seeded on glass coverslips, fixed with fixative solution for 15 minutes at room temperature and stained overnight at 37° C with the staining solution. Images were acquired by Zeiss microscope.

### FACS cytofluorimetry

WT and MDPL-HDFs were cultured and treated with cisplatin for 24 hours. After 24 and 48 hours from the end of treatment, cells were incubated for 6 hours with 10 μM BrdU (Life Technologies Corporation). Then they were collected, fixed with cold ethanol 90%, added with 1 mL of 2 N HCl + 0.5% Triton X-100 and resuspended with 1ml of 0,1 M borate buffer. Finally, pelleted cells were suspended with 200 μl of washing buffer + 1:50 mouse anti-BrdU antibody (Invitrogen, Life Technologies Corporation, CAT#33900, RRID: AB_86146) and incubated for 45 min at room temperature followed by two washes. Subsequently cells were incubated with FITC-conjugated secondary antibody (1:800) for 30 min at +4° C. For DNA content assay, cells were successively stained with propidium iodide (Sigma-Aldrich, St. Louis, MO, USA). Cytofluorimetric acquisition was done on a on a Gallios instrument using Kaluza G Acquisition software and the analyses were performed using Kaluza Analysis Software v.2.1 (all from Beckman Coulter, Inc.).

### Fractionation of fibroblast nuclei and Western blot

HDFs grown on a 100-mm dish were harvested with 0.05% trypsin-EDTA when they reached 70% confluence and rinsed with ice-cold PBS twice. Nuclei were separated from cytoplasm following the manual of NE-PER Nuclear and Cytoplasmic Extraction Reagents (#78835; Thermo Scientific). After centrifuging, the cytoplasm supernatant was removed. The pellets containing nuclei were resuspended in lysis buffer containing 50 mM Tris–HCl, pH 7.4, 150 mM NaCl, 1% Triton X-100, 0.1% deoxycholate, complete mini protease inhibitor cocktail, and subjected to slight sonication at 20% amplitude for 30 s. The whole nuclei lysate was further centrifuged at 16000 g for 5 min at cold. The supernatant was saved as the soluble fraction of the nuclei while the pellet was saved as the insoluble fraction of the nuclei. Both fractions were prepared for Western blot assay by adding Laemmli sample buffer (Bio-Rad). A one-fifth portion of either soluble or insoluble fraction sample was loaded onto 10% SDS–PAGE gel and then proceeded for Western blot analysis.

Nitrocellulose membrane was saturated in 5% milk/PBS and than probed with rabbit anti-Polδ NBP1-31541 (Novus Biologicals) and anti-Vinculin (EPR8185, Abcam), anti-NONO (A300-582A, Bethyl laboratories,), antibodies. Peroxidase-conjugated secondary antibodies were used (1:10000; EMD MilliporeCorporation, Billerica, MA, US). Signals were scanned and quantified on ImageQuant LAS 4000 system.

### Telomeric and genomic immunostaining of γH2AX

For X-ray experiments, HDFs at 12-13 PDL were used. Twenty-four hours before irradiation, 50,000 cells were seeded on slides inside 35-mm Petri dishes. Exposure to X-rays was performed in a 170 kV, 6 mA, 3mm Al filter, Gilardoni apparatus (Gilardoni, Italy) at a dose rate of 0.5 Gy/min. At different timepoints after irradiation, cells were fixed in 4% paraformaldehyde (PFA) for 15 min, permeabilized with 0.2% Triton-X and blocked in 1% BSA in PBS for 30 min at room temperature. Cells harvested at 4, and 24 hours following X-ray treatment were analysed by Western blot. Samples were co-immunostained over night at 4° C using mouse monoclonal anti-phospho-histone H2AX antibody (Millipore, CA, USA) in combination with a rabbit telomeric protein TRF1 antibody (Santa Cruz Biotechnology, CA, USA). After washes in 1% PBS/BSA, samples were incubated with secondary antibodies: Alexa 546-conjugated anti-mouse and Alexa 488-conjugated anti-rabbit (Invitrogen, CA, USA) for 1 h at 37° C. Finally, slides were washed in 1% PBS/BSA and were counterstained with DAPI and antifade solution. Interphase nuclei images were captured with 63X magnification with Axio-Imager Z2 fluorescent microscope, equipped with CCD camera (Metasystems, Milano, Italy). Frequencies of DNA damage foci γH2AX foci and TIF (co-localization spots with telomere protein and γH2AX) per cell were analyzed with ISIS software. Scoring analysis was performed on 100 nuclei for each experimental point.

### Collection of chromosome spreads and quantitative-fluorescence *in situ* hybridisation analysis (Q-FISH)

Chromosome spreads were obtained following 30 min incubation in 30 μM calyculin-A (Wako, Germany). Spreads of these prematurely condensed chromosomes (PCC) were prepared by a standard procedure consisting of treatment with a hypotonic solution (75 mM KCl) for 28 min at 37° C, followed by fixation in freshly prepared Carnoy solution (3:1 v/v methanol/acetic acid). The Q-FISH technique was based on the use of peptide nucleic acid (PNA) telomere oligonucleotides, that generate stronger and more specific hybridisation signals than the same DNA oligonucleotides. The Q-FISH allowed: (i) a precise measurement of individual telomeres at every single chromosome arm and (ii) to detect even small differences in telomere length [19]. Briefly, slides and probes (Cy3 linked telomeric and chromosome 2 centromeric Peptide Nucleic Acid PNA probes; PANAGENE, Korea) were co-denatured at 80° C for 3 min and hybridized for 2 h at room temperature in a humidified chamber. Slides were counterstained with 4,6-diamidino-2 phenylindole (DAPI, Sigma Aldrich, USA) in Vectashield antifade (Vector Laboratories, USA). Images were captured with 63X magnification with Axio-Imager Z2 fluorescent microscope equipped with a coupled charged device (CCD) camera (Metasystems, Milano, Italy). The telomere size was analysed with ISIS software (Metasystems, Milano, Italy), which calculates telomere lengths as the ratio between the total telomeres fluorescence (T) and the fluorescence of the centromere of the two chromosomes 2 (C). Data were expressed as a percentage (T/C%). Ten metaphases were scored for each experimental point.

### Statistical analysis

The difference between groups regarding IF and cellular analyses was tested by a paired Student t-test. Frequencies of γH2AX foci and TIF were analyzed with Mann–Whitney test. Values provided in the figures are means of three independent experiments ± standard deviation (SD). The level of significance was established at *P <0.05, ** *P*<0.01, *** *P*<0.001.

## Supplementary Materials

Supplementary Methods

Supplementary Figures
