# Supplementary material for: Functional analysis of POLD1 p.ser605del variant: the aging phenotype of MDPL syndrome is associated with an impaired DNA repair capacity
Source: Aging (Albany NY). 2021 Feb 22;13(4):4926–45. doi: 10.18632/aging.202680 (PMC7950258; doi:10.18632/aging.202680)
Supplement: Supplementary Methods [file aging-13-202680-s001.pdf]

SUPPLEMENTARY MATERIALS

Cell proliferation assay

For evaluating proliferation rate, WT and MDPL-HDFs (4 x 10^4 cells; p14) were seeded in each well of a 12 wells plate with DMEM (Dulbecco's Modified Eagle Medium; 10% FBS, 1% L-Glut (200x); 1% Pen/Strep (100x); Thermo Fisher Scientific). Then cells were detached with Trypsin treatment and counted with Burker chamber after 24 and 48 hours of culture at 37° C and 5% CO2.

Cisplatin treatment

For drug treatment cells were incubated with cis-Diammineplatinum (II) dichloride (Sigma Aldrich) [20µM] for 24 h at 37° C in a humidified atmosphere. For all the analyses cells were harvested before the beginning of the treatment (h0) and immediately after the 24 hour of cisplatin exposure (h24). Then they were collected and analysed 24 (+24h), 48 (+48h), 72 (+72h) and 96 (+96h) hours after drug removal.

Immunofluorescence assay

MDPL and WT-HDFs were fixed in 100% methanol at -20° C for 7 min or in 4% paraformaldehyde at RT for 10 min and incubated with the appropriate primary antibodies against Prelamin A (C-20; 1:100, Santa Cruz Biotechnology, INC), Lamin B1 (C-20; 1:100, Santa Cruz Biotechnology, INC), Lamin A/C (N-18; 1:100, santa Cruz Biotechnology, INC), phospho-Histone H2A.X (1:1000, Merck Millipore), Pold1 (1:200, Novus Biologicals), LC3B (NB100-2220-Novusbio) and LAMP1 (MAB 48000 R&D Systems). Appropriate Alexa Fluor 488- or 568-labeled secondary antibodies were incubated for 1 h at room temperature (Invitrogen, Carlsbad, CA, USA). Nuclei were labeled with 4,6-Diamidino-2-phenylindole (DAPI-Sigma Aldrich) and examined under a fluorescence microscope. Images are acquired using a Zeiss (Zeiss, Thornwood, NY, USA) Axioplan 2 microscope. Immunofluorescence analyses were conducted from passage 10 to 15.
