# Supplementary material for: Functional analysis of POLD1 p.ser605del variant: the aging phenotype of MDPL syndrome is associated with an impaired DNA repair capacity
Source: Aging (Albany NY). 2021 Feb 22;13(4):4926–45. doi: 10.18632/aging.202680 (PMC7950258; doi:10.18632/aging.202680)
Supplement: Supplementary Figures [file aging-13-202680-s002.pdf]

SUPPLEMENTARY FIGURES

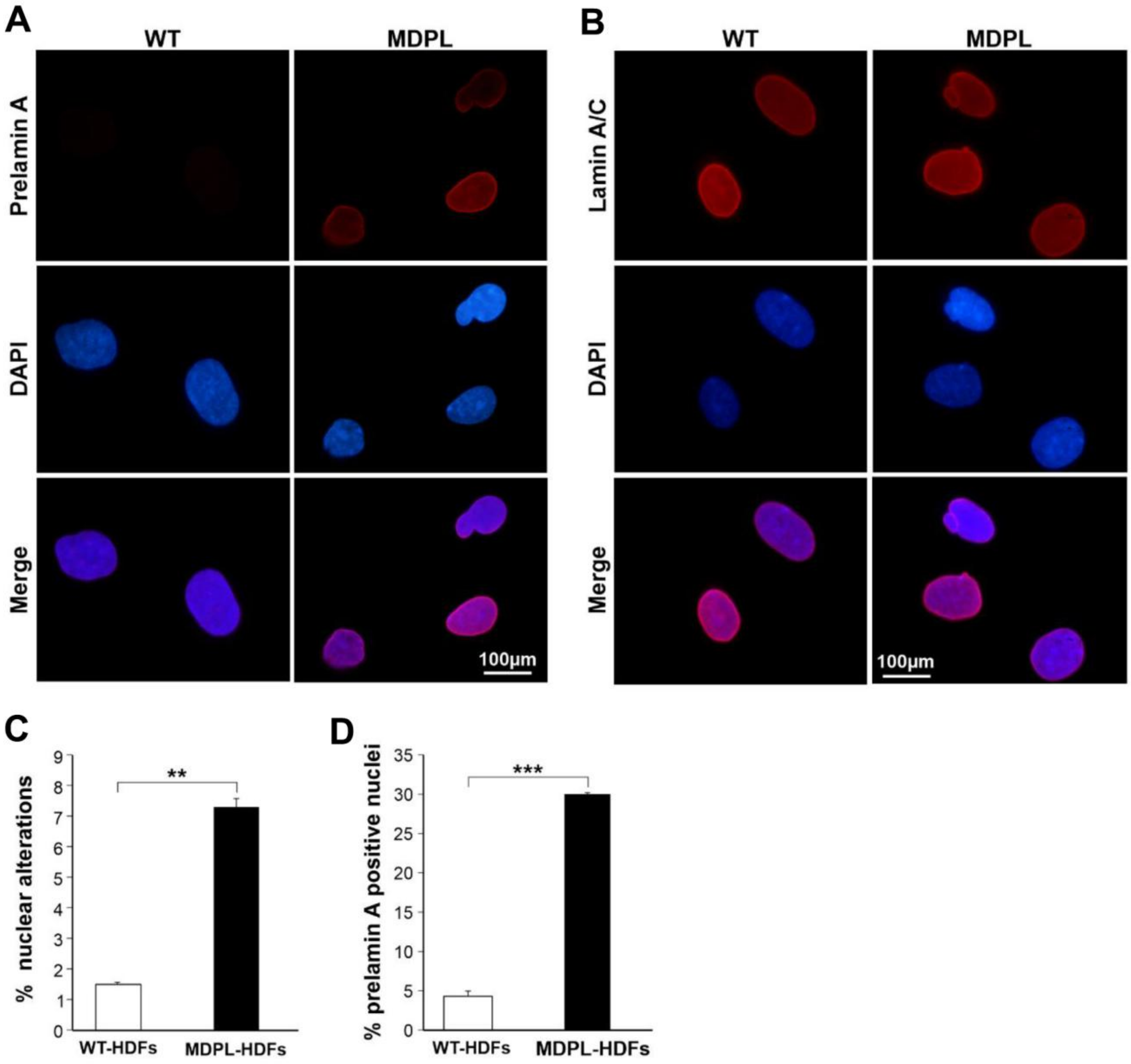

Supplementary Figure 1. (**A**, **B**): Representative images of aberrant nuclear envelope conformations highlighted with DAPI staining. Immunofluorescence analyses show the abnormal presence of prelamin A (red) in MDPL-nuclei, while control cells (WT) show regular nuclear envelope shape and a rarely detection of prelamin A staining. DAPI nuclear staining (blue). Scale bar 100µm. (**C**): Histogram representing the percentage of aberrant nuclear conformations. Error bars represent the SD from the analysis of 100 cells from three independent experiments and WT values are displayed as the average percentages of 2 different controls (**P<0.01). (**D**): Histogram representing the percentage of prelamin positive nuclei. Error bars represent the SD from the analysis of 100 cells from three independent experiments and WT values are displayed as the average percentages of 2 different controls (***P<0.001).

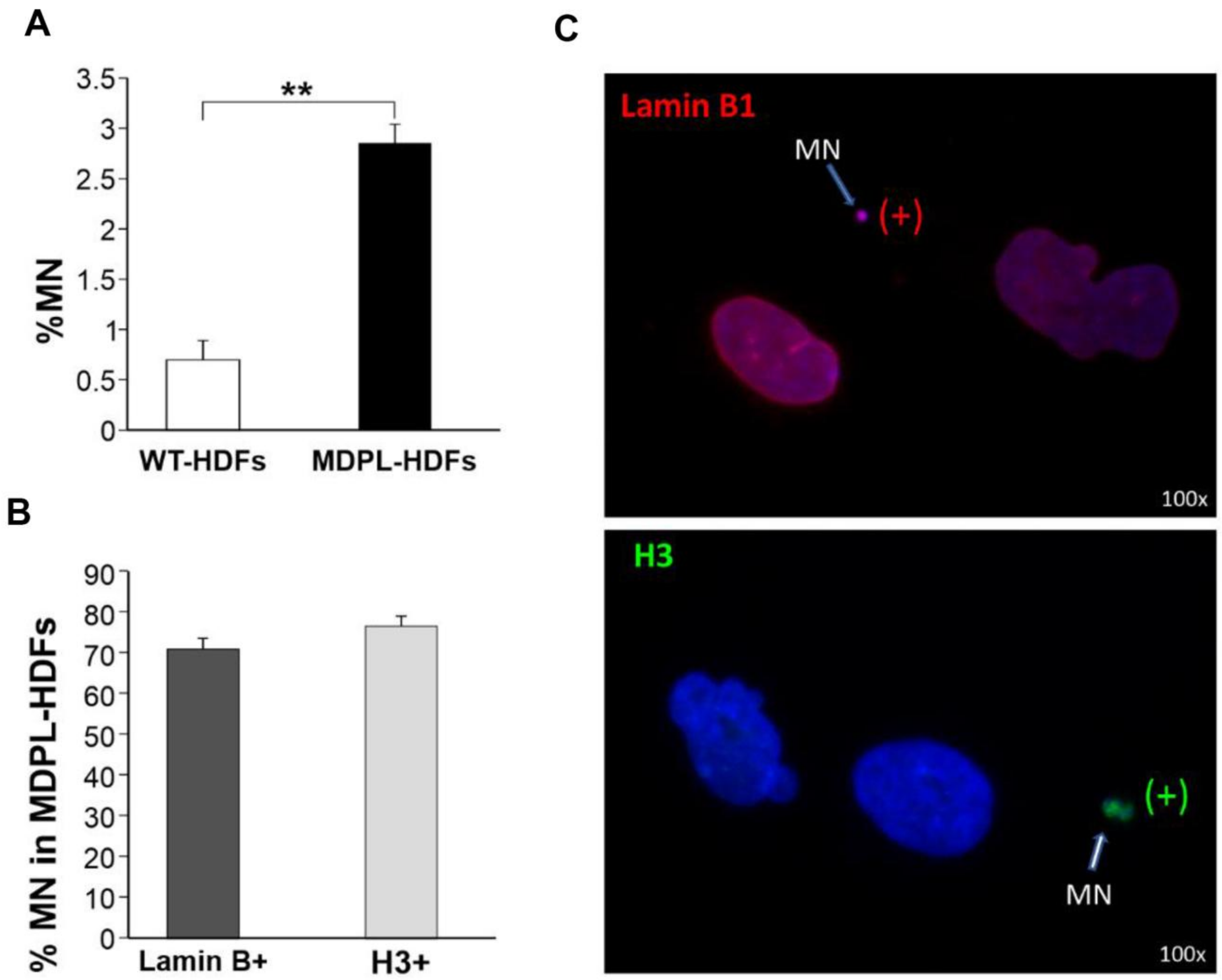

Supplementary Figure 2. (**A**) The graph shows the percentage of micronuclei encountered in WT and MDPL-HDFs. The data have been obtained counting micronuclei after DAPI staining for fluorescence imaging. Error bars represent the SD from the analysis of 100 cells from three independent experiments and WT values are displayed as the average percentages of 2 different controls (***P*< 0.01). (**B**): The histogram shows the percentages related to Lamin B1 and Histone H3-positive micronuclei in MDPL HDFs. Error bars represent the SD from the analysis of 100 cells from three independent experiments. (**C**): Representative immunofluorescence images showing the presence of micronuclei (MN) positive for Lamin B1antibody (red) and Histone H3 antibody (green) in MDPL cells. DAPI nuclear staining (blue). Scale bar 100µm.

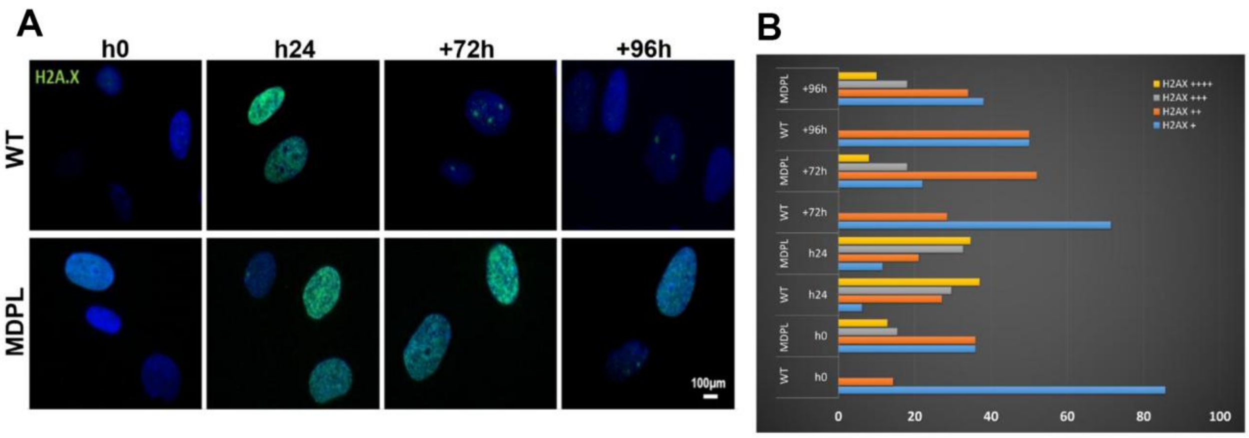

Supplementary Figure 3. (**A**): Representative image of immunofluorescence detection of γH2A.X foci. WT and MDPL HDFs were exposed to cisplatin drug for 24 h, fixed after 72 (+72h), 96 (+96h) hours from the end of cisplatin treatment and stained with a monoclonal Anti-phospho-Histone H2A.X antibody. (**B**): Quantitative analysis of γH2A.X foci positive signal. (+) and (++) were defined as low expression, while (+++) and (++++) were defined as high intensity of immunofluorescence signal. WT values are displayed as the average percentages of 2 different controls.

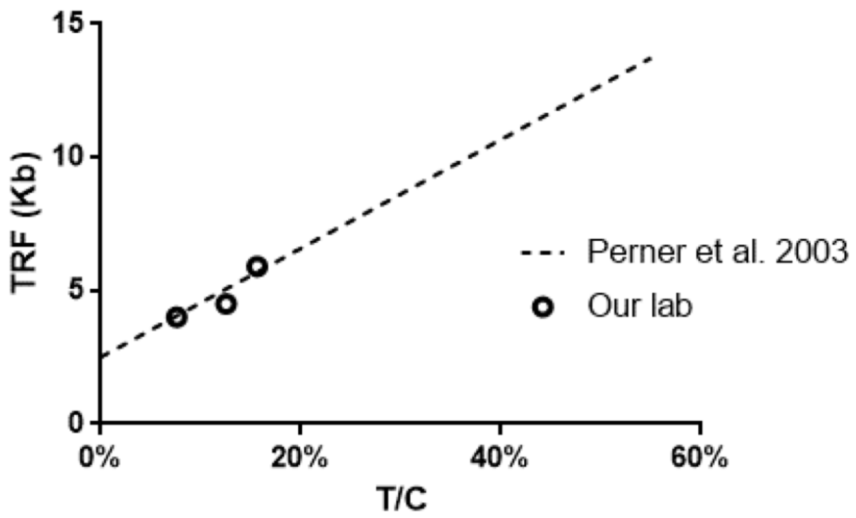

Supplementary Figure 4. Relative telomere lengths were evaluated as the ratio between the total telomeres fluorescence (T) and the fluorescence of the centromere of the two chromosomes 2 (C), giving the value T/C. In order to convert them in absolute telomere length, expressed in kilobases (kb), we used the formula and data of Perner et al. (2003). In order to confirm and calibrate with our procedures, we used 3 tumor cell lines (U251, DU 145 and MCF-7) of our laboratory, for which we know their telomere length, and measured their relative telomere length (T/C). As shown in figure our data fitted very well with those expected by the regression line of Perner et al. (2003). Therefore, we used the formula of Perner et al. (2003): *Absolute telomere length (in kb) = 2.507 + 0.204 * (T/C)* to convert T/C values of the fibroblasts under study.
